# Media screen time use and mental health in school aged children during the pandemic

**DOI:** 10.1186/s40359-023-01240-0

**Published:** 2023-07-10

**Authors:** Amira Hmidan, Diane Seguin, Emma G. Duerden

**Affiliations:** 1 Applied Psychology, Faculty of Education, Western University, 1137 Western Rd, London, ON N6G 1G7 Canada; 2 Department of Psychology, Western University, N6A 3K7 London, Canada; 3 Physiology & Pharmacology, Schulich School of Medicine and Dentistry, Western University, London, Canada; 4 Children’s Health Research Institute, London, Canada

**Keywords:** Parent stress, Parental involvement, Screen time, Behavioural outcomes, Education

## Abstract

**Background:**

Children’s screen time activity has increased significantly during the pandemic. Extended school closures and heightened parent stress are associated with children’s behavioural difficulties and time spent watching screens. The primary aim of this study was to determine which school and household factors were associated with challenging behaviours in Canadian schoolchildren during the COVID-19 pandemic.

**Methods:**

This longitudinal survey study examined the association amongst screen time, internalizing and externalizing behaviours in school-aged children at two time points over the 2020–2021 academic school year. Parents completed survey measures on their parental involvement, stress levels, and their child’s screen time use as well as their emotional and behavioural difficulties.

**Results:**

Children’s average daily screen time was 4.40 h (*SE* = 18.45) at baseline and 3.89 h (*SE* = 16.70) at 1-year follow up, with no significant change across the school year (*p* = .316). Increased screen time use was associated with a greater incidence of internalizing behaviours in children (*p* = .03). Children who spent more time on screens and who were in households with parents reporting higher stress levels had increased internalizing behaviours (*p* < .001). No association between screen time use and externalizing behaviours was evident; however, parent stress was positively associated with children’s externalizing behaviours (*p* < .001).

**Conclusions:**

Children’s screen time use has remained high during the pandemic and is associated with anxious and depressive symptoms. Children who spent more time on screens and who were in households with parents reporting higher stress levels had increased internalizing behaviours. Parent stress was positively associated with children’s externalizing behaviours. Targeted family intervention plans focused on reducing parent stress and screen time use may aid in improving children’s mental health during the ongoing pandemic.

## Introduction 

The COVID-19 pandemic generated unprecedented increases in children and adolescents’ time spent on devices, with some children spending over 6 h per day on screens [1–4]. A parallel epidemic emerged in parents with school-aged children, who reported stress levels in the moderate to high range [3, 5, 6]. While screen time and parent stress are known to have separate adverse implications on childhood development [7–9], little is known about the accumulated risks of prolonged screen time exposure on incidences of internalizing and externalizing behaviours in children, and the moderating influence of parent stress and parenting strategies on these relationships.

Screen time is the amount of time spent using a device with a digitized interface, such as computers, television, video games, and smartphones [10]. Health care professionals caution that excessive screen time may adversely impact childhood development, health, and mental health outcomes with screen time guideline recommending a maximum of 2 h of screen time per day for children over the age of five [11, 12].

The unparalleled impact of COVID-19 engendered nationwide increases in children’s time spent on devices [1, 4, 13, 14]. Based on early cross-sectional data in Ontario, children’s average screen time increased from 2.6 h before the pandemic, to 5.9 h a day during the initial school closures of the pandemic [3]. This daily average is more than double the recommended guidelines for Canadian children. Similar increases occurred in the United States, Turkey, China, Italy, and Spain [1, 2, 4], with concurrent decreases in physical activity also found in some of these samples [4, 13]. Factors such as socioeconomic status, race/ethnicity, school satisfaction (for both children and parents), and having a child with special needs, contributed to unregulated screen time in children [2, 3, 15, 16].

The most prevalent mental health concerns affecting school-aged children prior to the pandemic were anxiety, depression, and conduct problems [17]. Since March 2020, internalizing (i.e., anxiety and depression) and externalizing problems (i.e., aggression) have increased dramatically in school aged children [18], with an estimated 20–25% of children experiencing symptoms of depression and anxiety [19]. Lockdowns were associated with the most pervasive emotional problems, including anxiety, restlessness, worry, and depression [13, 19].

Early evidence indicates that internalizing and externalizing problems may be an enduring issue for children and youth during the pandemic. Increases in internalizing and externalizing symptoms from pre-pandemic to the initial lockdown period were found in Canada and the United Kingdom [18, 20]. In a cohort of Norwegian children, an overall increase in mental health issues were reported during the early stages of the pandemic to 9 months after the initial outbreak, with internalizing symptoms accounting for the most increases [21]. Factors such as parent stress, intra-family dysregulation, and screen time contributed to increases in children’s mental health problems [6, 18, 22].

Excessive screen time use appears to have an adverse influence on children’s mental health outcomes [23–25]. In a large longitudinal study of brain development and child wellbeing in the United States (ABCD study; https://abcdstudy.org) increased screen time was associated with impaired social skills [26, 27], depression and anxiety [28], behavioural and social issues [29], and reduced sleep duration, fatigue, and insomnia in children [30]. Screen time was associated with a dose-dependent increase in depression risk in children [31]. Youth reporting over 4 h of passive screen time per day were significantly more likely to meet the criteria for major depressive episode, social phobia, and generalized anxiety disorder [32]. By contrast, a systematic review (*n* = 159, 425) on children’s screen time behaviours found that screen time was weakly associated with externalizing and internalizing problems [33]. Age-related factors appear to influence the strength and direction of the association [34].

Adverse mental health outcomes associated with unregulated screen time is an emergent concern amid the ongoing pandemic. Cross sectional evidence from early stages of the pandemic demonstrates associations between excessive screen time and increased incidence of total mental health difficulties in children and youth, with sleep duration, physical activity and sedentary behaviour mediating these relationships [25, 35]. In a cohort of 2026 Ontario children, more digital media and TV consumption was associated with conduct and hyperactivity/inattention problems in children under the age of 4 and internalizing problems in older children [36].

Parent mental health, parenting strategies, and involvement with children’s academic and recreational activities may mediate the association between excessive screen time use and mental health problems in children [3, 10, 37–39]. For example, in an Israeli cohort of parent-adolescent dyads, negative parenting practices significantly increased the risk of digital media addiction in children, which in turn was associated with behavioural and emotional problems [40]. In a Brazilian cohort, negative parenting strategies were associated with both poorer mental health in children, as well as increased screen time [22].

The pandemic has contributed to heightened perceived stress among parents with school aged children [3, 5, 13, 41], which may put children at risk for greater screen time use and mental health problems in the future [6, 42]. For example, greater parent anxiety during the initial COVID-19 outbreak was a significant predictor of children’s internalizing and externalizing behaviours [6]. In addition, over-reactive, inconsistent, and authoritarian parenting practices contributed to higher screen time use in children [1, 22].

While studies point to the separate negative influences of screen time and parent stress on children’s mental health outcomes, few studies have addressed these concerns longitudinally. The influence of parental stress and parenting strategies has been identified as important mediating factors on both screen use behaviours in children as well as emotional and behavioural outcomes. It is essential to identify the factors that promote or impede childhood development, and to devise early intervention strategies to support parents and children during periods of school closures.

In the current longitudinal study, we examined the association between screen time, parental involvement, and parent stress on the incidences of externalizing and internalizing behaviours in a cohort of Canadian children from November 2020 to November 2021. These time points encompass different school and public responses to COVID-19. In late 2020 to early 2021, much of Canada was under lockdown measures, resulting in school closures for in-person learning, and the closure of community and recreational centres. In late 2021, all adults were eligible for vaccination, schools across the country were open for in-person learning, and community and recreational centres were resuming activities. Using data gathered from these diverse time points, two research questions were examined: 1) Is screen time associated with internalizing and externalizing behaviours in children over time? 2) Will changes in parent stress and parental involvement affect the relationship between screen time and children’s mental health outcomes? We hypothesize that protracted screen time exposure will contribute to increased externalizing and internalizing behaviours, and that parent stress and parental involvement will moderate the strength of this association.

## Methods

### Participants 

We obtained data from an ongoing longitudinal community-based cohort project on children’s mental health and learning outcomes during the pandemic (CoMPASS: COVID-19 Managing Parent Attitudes and School Stress). Parents (aged 18 years and over) and children (aged 6–12 years) residing in Canada with children receiving education through the public-school systems were invited to participate in this study. Participants were recruited online through Prolific and social media (i.e., Facebook, Twitter, and Instagram). Parents provided informed consent/assent. The study received approval from the Non-Medical Research Ethics Board at Western University.

### Procedures

Data from two time points across the 2020–21 academic school year were considered for the present study. Baseline data were collected from parents between November 2020 and April 2021 on a rolling recruitment basis. Parents completed follow up surveys approximately 12 months from their baseline measures (June 2021- November 2021). Parents completed demographic measures and answered questions about their parental involvement, stress levels, and their child’s mental health and screen time behaviours. We condensed the demographics questionnaire at the second time point and incorporated supplementary items to capture pandemic-related alterations to school and home routines. All other procedures remained the same.

### Demographic measures 

Parents provided information regarding their household income, employment status, age, gender, geographical location, and the number of children living at home. Children’s information, including age, special needs and mental health diagnoses, and mode of education, was provided by the participating parent.

### Parent measures

Parental involvement was assessed using the parental involvement subscale from the abbreviated Alabama Parenting Questionnaire [43]. The APQ is a well-documented tool comprised of 42 items that provides a score for 5 subscales, one of which were used for this analysis: parental involvement captures how engaged the parent is in their child’s learning. For each item parents indicate how often they engage in the behaviour on a 5-point scale, with responses ranging from Never (value of 1) to Always (value of 5). Scores are summed for each subscale, with 50 points possible for parental involvement. The Cronbach’s α reliabilities were reported for the APQ and the ranges for parent involvement for these values were 0.77 to 0.82 [43, 44].

The Parent Stress Index-Fourth Edition Short Form (PSI-4) [45] was used to assess the magnitude of stress experienced by parent–child dyads. The PSI-4 contains three 12-item subscales, including Parental Distress, Parent–Child Dysfunctional Interaction, and Difficult Child. Each item is answered using a 5-point scale, with responses ranging from Strongly Disagree (value of 1) to Strongly Agree (value of 5). Subscales were summed to create the Total Stress Index. Reliability was assessed with test–retest indicators ranging from 0.68 to 0.91 [46].

### Child measures 

We assessed recreational screen time use through parent estimates of time spent on social media, video gaming, and watching television. Parents provided estimates for sleep duration, time spent engaged in physical activity, and time spent on homework. Time-based estimates were measured in minutes per day. Parents indicated whether their child was enrolled in online or in-person schooling, as well as the parent’s satisfaction with their child's education and whether their child was engaged in their schoolwork.

We examined children's internalizing and externalizing behaviours through the Strengths and Difficulties Questionnaire (SDQ) [47]. The SDQ is a validated behavioural questionnaire for caregivers of children (3–16 years old). The SDQ utilizes five subscales to assess emotional and behavioural development (i.e., emotional symptoms, peer relationship problems, conduct problems, hyperactivity–inattention, and prosocial behaviour). Parents respond with how true each item is in regards to their child’s behaviour over the past 6 months. An internalizing subscale is composed of scores for peer relationships and emotional difficulties scores. An externalizing subscale is composed of conduct problem and hyperactivity/inattention scales, while prosocial scores form the prosocial subscale [47]. The internalizing and externalizing subscales are summed to produce a total difficulties score, with greater total difficulty scores significantly associated with greater prevalence of clinical diagnoses [47].

### Statistical analyses 

All statistical analyses were performed using IBM SPSS Statistics software (Version 28, Statistical Package for the Social Sciences, IBM, Armonk, NY). A multiple imputation procedure addressed missing values in the dependent variables. We performed an analysis of missing values and Little’s MCAR test to determine whether missing data were missing completely at random (MCAR). Missing values were imputed using predictive mean matching.

Our first aim was to examine whether screen time was associated with children’s mental health difficulties. Generalized estimating equations models (GEE) with an identity link function were used for the analyses. In the initial step of the GEE, screen time (measured in minutes) was entered as a continuous predictor variable with internalizing and externalizing behaviours entered as dependent variables in separate models. Time was included as a within-subjects factor to account for repeated measures. 

Our second aim was to determine whether parent stress and parental involvement modified the relationship between screen time and mental health outcomes. This was addressed by introducing parent stress and parental involvement into the models as covariates.

All models were adjusted for family income, number of children living at home, sleep, physical activity, and special needs. Parent gender may influence subjective reports of children’s mental health [48] and in turn this was entered as a covariate in all models. Additionally, parent ratings of children’s behaviour may also be influenced by the age of the children, and in turn this variable was also included as a covariate in the analysis.

## Results 

### Participant characteristics

A total of 210 parents completed the questionnaire at T1 (baseline). After excluding ineligible participants and those missing data, a total of 193 participants were retained for the study and participants completed measures at the one year follow up (*n* = 113) prior to data cleaning. Participants who completed measures at both time points (51%, *n *= 108) were included in the final analysis. Missing data for the final sample ranged from 3–5 data points for some of the dependent and independent variables. Missing values were imputed using predictive mean matching. No significant differences in parent gender, age, child age or income levels were evident between those individuals who participated in the baseline assessments and the follow up time point 1 year later (all, *p *> 0.30). The median age was 38.00 (*IQR* = 8.00) years for parents and 8.00 (I*QR* = 3.00) years for children. Participant demographics are presented in Table 1.

**Table 1:** Participant demographics

Characteristic	Full sample		
	*N*	%	Mean (SD)
Parent Gender			
Women	56	52.8	
Men	50	47.2	
Parent age groups			37.54(6.62)
19–29	11	10.5	
30–39	56	53.3	
40–49	35	33.3	
50–59	3	2.9	
Primary parent^a^	102	96.2	
Cohabitate^a^	99	94.3	
Number of children at home			1.94(.93)
1–2	82	78.1	
3–4	21	20	
5 +	2	1.9	
Income			$101,450(447.80)
Low < 40 K	7	7.1	
Moderate 40- 80 K	29	29.6	
High > 80 K	62	63.3	
Educational level			
Highschool	6	5.7	
College/some college	22	21.0	
University/some university	54	51.4	
Masters/postgraduate	18	17.1	
Doctorate	5	4.8	
Employment^a^	93	12.3	
Spouse Employment^a^	80	85.1	
Child age groups			8.52(2.10)
6 – 7	39	39.4	
8 – 9	27	27.3	
10 – 12	33	33.3	
School delivery			
Online	40	37.7	
In-person	66	62.3	
School enrollment			
Full-time	103	98.1	
Part-time	2	1.9	
Child with special needs^a^	11	10.2	

^a^Reflects the number and percentage of participants answering “yes” to this question

### Screen time and child mental health 

The average time children spent on screens was 252.20 min (4.40 h; *SE* = 18.45) at T1 (baseline) and 233.99 min at follow up 1-year later (3.89 h; *SE* = 16.70). No significant differences in daily screen time were evident between the two time points (*t*(103) = 1.01, 95% C.I. [-17.63 – 54.05], *p* = 0.316). The average score for internalizing behaviours was 4.20 (SD = 3.05) at baseline and 4.09 (SD = 3.57) at follow up, while the average externalizing behaviour score was 5.32 (SD = 3.55)) at baseline and 5.31 (SD = 3.40) at follow up. No significant differences were found for internalizing (*t*(104) = 0.652, 95% C.I. [-0.408 – 0.808], *p* = 0.516), or externalizing behaviours (*t*(104) = 0.096, 95% C.I. [-0.561 – 0.619], *p* = 0.924) between the two time points.

### Parental factors 

Parent stress levels increased significantly from baseline (*M* = 78.45, *SE* = 1.89) to the second time point ([*M* = 86.23, *SE* = 2.59], (*t*(104) = -4.59, 95% C.I. [-11.13 – -4.41], *p* < 0.001). The average score for parental involvement was 38.35 (SD = 5.82) at baseline and 38.10 (SD = 6.231) at follow up. No significant differences were found in parental involvement scores between time points (*t*(104) = 0.758, 95% C.I. [-0.508 –1.14], *p* = 0.450).

### Internalizing behaviours and screen time 

A significant positive main effect for screen time and internalizing behaviours was evident (*p* = 0.030, Table 2: model 1). Children’s physical activity levels (*p* = 0.001) and older ages in the children (*p* = 0.027) were associated with decreased internalizing behaviours.

**Table 2:** GEE regression model for children’s internalizing behaviours

	**Model 1**					**Model 2**					**Model 3**				
	*B*	*SE*	95%	CI	*p*	*B*	*SE*	95%	CI	*p*	*B*	*SE*	95%	CI	*p*
			*LL*	*UL*				*LL*	*UL*				*LL*	*UL*	
Parent gender^a^	-.590	.55	-1.67	.487	.283	-.577	.468	-1.49	.340	.218	-.548	5.30	-1.59	.490	.301
Special needs^b^	-1.94	1.00	-3.89	.015	.052	-2.24	.810	-3.82	-.648	.006*	-2.06	.931	-3.89	-.237	.027*
Income	.002	.007	-.011	.015	.738	.000	.006	-.011	.012	.959	.002	.006	-.011	.014	.768
Children at home	.450	.265	-.069	.970	.089	.479	.202	.084	.875	.017*	.508	.245	.028	.988	.038*
Child age	-.255	.115	-.481	-.029	.027*	-.172	.100	-.361	.017	.075	-.265	1.06	-.472	-.059	.012*
Physical activity	-.008	.002	-.012	-.003	.001**	-.006	.002	-.010	-.002	.007*	-.007	.002	-.012	-.003	.001**
Sleep	.001	.004	-.007	.009	.806	.002	.003	-.005	.009	.570	.003	.004	-.005	.010	.482
Screen time	.003	.001	.000	.006	.030*	.002	.001	.000	.005	.036*					
PSI^c^						.068	.010	.048	.088	.001**					
Parental Involvement						.086	.041	.006	.116	.034*	.007	.035	-.062	.076	.840
PSI*Screen time											< .001	0.0001	0.000001	0.0001	.001**

^a^0 = Female; 0 ^b^ = No developmental disorder; ^c^ Parent stress index. * *p* < .05, **p < .001. Model 1: A significant positive main effect was found for screen time and internalizing behaviours (*p* = .030). Model 2: Children’ screen time was further explored in an extended model including parent stress and parental involvement, which were both significantly associated with children’s internalizing behaviours (both, *p* < .05). Model: 3 An interaction model with screen time and parent stress were significantly associated with one another (*p* = .001)

### Internalizing behaviours, screen time, and parental factors

In an extended model (model 2), screen time and parent stress (PSI) were positive predictors of internalizing behaviours (both, *p* < 0.05, Table 2: model 2). Greater parental involvement, and number of children at home were also positively associated with internalizing behaviours (both, *p* < 0.05). Decreased levels of physical activity was associated with fewer internalizing behaviours.

**Fig. 1:**
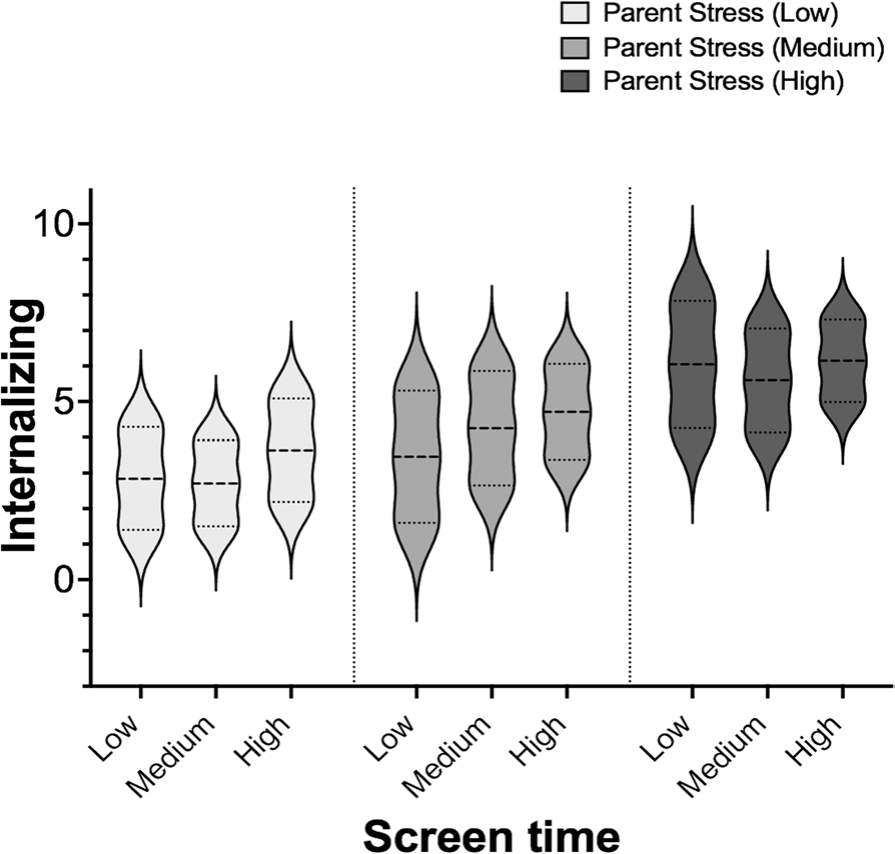
The interaction effects of screen time behaviour and parent stress on internalizing behaviours. Parent stress and screen time were transformed into grouping variables based on quartile ranges (Low, < 25^th^; Medium; < 50^th^, High, > 75^th^ percentile). Scores for internalizing behaviours remained continuous. A significant interaction was found for parent stress and screen time activity (β =  < .001, *p* < .001). Scores represent estimated marginal means with 95% Wald Confidence Intervals

In an interaction model (Table 2: model 3), increased internalizing behaviours were exhibited by children who had elevated screen time and who had parents reporting high stress, with the lowest internalizing behaviour scores reported for children with less screen time and who had parents reporting lower stress (*p* < 0.001, Fig. 1). Children’s physical activity and age of the child were negatively associated with increased internalizing behaviours, while number of children at home was a significant positive predictor in the model (all, *p* < 0.05). Parental involvement did not significantly modify the relationship with internalizing behaviours in this model (*p* > 0.05).

### Externalizing behaviours and screen time and parental factors

No association was found between screen time and externalizing behaviours after adjusting for covariates in the GEE model (*p* > 0.05). When parent stress and parental involvement were included in the model as moderating variables parent stress was a positive predictor of externalizing behaviours (β = 0.43, 95% C.I. [0.021-0.066], *p* < 0.001). No other significant results were found.

## Discussion 

The current study explored whether children with higher screen time use would exhibit more externalizing and internalizing behaviours than children with lower screen time use. We also considered the moderating influence of parental stress and involvement on behavioural outcomes and univariate changes to screen time, parenting factors and child mental health.

Children's screen time soared during the initial wave of the pandemic, with several North American and European countries reporting estimates of over 6.00 h per day [1–4]. While we found no significant changes in screen duration over the study period, children were spending an average of 4.00 h per day on screens, which remains higher than pre-pandemic levels [3, 49] and exceeds the recommended guidelines for Canadian children [11, 12]. This finding illustrates that continuous screen exposure over protracted periods may still pose health risks to children and adolescents.

Parent stress levels were within normal limits at both time points; however, there was a significant 7-point increase at the 1-year follow-up from baseline. This finding corresponds with, and extends upon, previous observations of parents' perceived stress amidst the pandemic. Previously, parents reported experiencing high-stress levels at the outset of the pandemic [3, 5, 41], with stress levels waning toward the end of 2020 [41]. We found an increase in perceived stress toward the end of 2020 and into 2021, suggesting that parents continue to struggle with competing demands presented at home and in the workplace.

### Internalizing behaviours and screen time 

Children's internalizing behaviours were outcomes of interest in the current study. We found a moderate positive association between screen time and internalizing behaviours after controlling for demographic covariates, sleep duration, and physical activity. This observation corresponds with Eirich and colleagues’ (2022) meta-analysis demonstrating weak associations between screen time and children's internalizing and externalizing behaviours [33]. Outcome heterogeneity was moderated by between-study variation.

Age-related factors could moderate the correlational strength of screen time and internalizing behaviours. For example, Neville and colleagues (2021) found that greater screen time among preschoolers significantly predicted internalizing behaviours at ages 5 and 7, but greater screen time at age 7 was significantly associated with less internalizing behaviours at 9 years of age [34]. Screen time may disproportionately influence internalizing symptoms in children depending on their age [36]. Therefore, the modest association found in the current work may reflect the association between media use and internalizing symptoms in school-aged children. Nevertheless, screen time overexposure (≥ 4.00 h per day) puts children at a threefold risk for developing major depressive disorder, social phobia, and generalized anxiety disorder [32], which underscores the necessity of limiting children's screen time activity, irrespective of age.

### Internalizing behaviours, screen time, and parental factors

Parent stress and parental involvement modified the influence of screen time on internalizing behaviours. We also found evidence of a strong relationship between screen time and parent stress on the incidence of anxiety and depressive symptoms in children. Previous research demonstrates a strong positive association between parent mental health and screen time behaviours, which is mediated by dysfunctional parent–child interactions, inconsistent screen time monitoring [10], limited access to childcare resources [42], lower household income [3, 16], extended school closures [16], and negative parenting strategies [22]. Altogether, our findings indicate that children with greater screen time engagement and who dwell in high-stress environments are at significant risk of developing internalizing behaviours.

### Externalizing behaviours and screen time 

Our second outcome of interest was externalizing behaviours, which were comprised of hyperactivity/inattention symptoms and conduct problems. We found no association between screen duration and externalizing behaviours in school-aged children. Previous studies indicate that externalizing issues emerge more frequently in younger children. For example, Neville and colleagues (2021) found a directional association between externalizing behaviours and screen time activity in preschoolers, suggesting that parents may use technology to regulate challenging behaviour exhibited by young children [34]. Spending over 2 h per day on screens was found to increase preschoolers’ risk for developing inattention issues by the age of 5; however, the association with aggressive behaviours was not apparent [24]. Overall, screen time and externalizing difficulties appear weakly correlated [33], suggesting that other latent factors may underpin children's behavioural functioning.

### Externalizing behaviours, screen time, and parenting factors 

Parent stress emerged as a strong predictor for children's externalizing behaviours over the pandemic. The association between child behavioural difficulties, parent–child dysfunctional interactions, and parent stress is well established in the literature [22, 38, 39]. Parent stress is associated with externalizing behaviours in adolescents, which underscores the need to address parenting strategies and parent mental health when treating children with externalizing difficulties [33, 39].

### Limitations 

A limitation of the current study was the use of online survey questionnaires to collect information. Albeit virtual collection methods attenuate pandemic-related bottlenecks presented in research, the lack of in-person observation exacerbates the risk of systemic and random error. As we recruited from social media, our sample is biased towards parents who use social media and may be more comfortable using technology. This may bias some responses related to the ease with which parents were able to access online information and platforms for their children’s online learning, or school/teacher communication methods. A strength of our study was the compatibility with multiple technologies (i.e., tablets, smartphones, and laptops) and accessible to English and French speaking families. Another theoretical limitation of the current work is that children’s outcome data were exclusively derived from parent-report estimates. Children often have disparate perceptions of their school and home relationships, which may differ from parental reports. Finally, our study had modest attrition rates.

## Conclusion

The current study provides insight to the factors that promoted or impeded children’s emotional and behavioural outcomes over the 2020–2021 academic school year; and the influence of parent stress and parenting. Screen time usage in children increased during the initial phases of the pandemic and our current longitudinal study found elevated screen usage persisted in children following a year of pandemic life. Increased screen use in children and high parental stress were associated with increased anxious and depressive symptoms in children, with the highest scores for these internalizing behaviours seen in children who had both elevated screen use and parents experiencing high stress. Future studies are needed to investigate the long-term effects of pandemic life on children's development and identify whether the associations we report remain as pandemic restrictions ease. Additionally, future research should examine modifiable home and parental factors which contribute to internalizing behaviours in children to aid in targeted treatments for anxiety and depression, and inform parental education programs.

## Data Availability

The datasets used and/or analysed during the current study available from the corresponding author on reasonable request.

## Abbreviations

APQ: Alabama Parenting Questionnaire
PSI: Parent Stress Index -Fourth Edition Short Form

## References

[1] Eyimaya AO, Irmak AY (2021). Relationship between parenting practices and children’s screen time during the COVID-19 pandemic in Turkey. J Pediatr Nurs.

[2] Nagata JM (2022). Screen Time use among US adolescents during the COVID-19 pandemic: findings from the Adolescent Brain Cognitive Development (ABCD) Study. JAMA Pediatr.

[3] Seguin D, Kuenzel E, Morton JB, Duerden EG (2021). School’s out: Parenting stress and screen time use in school-age children during the COVID-19 pandemic. J Affect Disord Rep.

[4] Xiang M, Zhang Z, Kuwahara K (2020). Impact of COVID-19 pandemic on children and adolescents’ lifestyle behavior larger than expected. Prog Cardiovasc Dis.

[5] Calvano C (2021). Families in the COVID-19 pandemic: parental stress, parent mental health and the occurrence of adverse childhood experiences—results of a representative survey in Germany. Eur Child Adolesc Psychiatry.

[6] Li X, Zhou S (2021). Parental worry, family-based disaster education and children’s internalizing and externalizing problems during the COVID-19 pandemic. Psychol Trauma Theory Res Pract Policy.

[7] Madigan S, Browne D, Racine N, Mori C, Tough S (2019). Association Between Screen Time and Children’s Performance on a Developmental Screening Test. JAMA Pediatr.

[8] Lissak G (2018). Adverse physiological and psychological effects of screen time on children and adolescents: Literature review and case study. Environ Res.

[9] Osofsky JD, Thompson MD. Adaptive and Maladaptive Parenting: Perspectives on Risk and Protective Factors. In Handbook of Early Childhood Intervention. Shonkoff JP, Meisels SJW, editors. Cambridge University Press; 2000. p. 54–75 10.1017/CBO9780511529320.005.

[10] Tang L, Darlington G, Ma DWL, Haines J (2018). Mothers’ and fathers’ media parenting practices associated with young children’s screen-time: a cross-sectional study. BMC Obes.

[11] Canadian Paediatric Society, Digital Health Task Force, Ottawa, Ontario (2019). Digital media: promoting healthy screen use in school-aged children and adolescents. Paediatr Child Health.

[12] World Health Organization. WHO guidelines on physical activity and sedentary behaviour: at a glance. Geneva: World Health Organization; 2020.

[13] Orgilés M, Morales A, Delvecchio E, Mazzeschi C, Espada JP (2020). Immediate Psychological Effects of the COVID-19 Quarantine in Youth From Italy and Spain. Front Psychol.

[14] Velde G (2021). Physical activity behaviour and screen time in Dutch children during the COVID -19 pandemic: Pre-, during- and post-school closures. Pediatric Obes.

[15] Assari S (2020). American Children’s Screen Time: Diminished Returns of Household Income in Black Families. Information.

[16] Lee S (2022). Relationship between screen time among children and lower economic status during elementary school closures due to the coronavirus disease 2019 pandemic. BMC Public Health.

[17] Ghandour RM (2019). Prevalence and Treatment of Depression, Anxiety, and Conduct Problems in US Children. J Pediatr.

[18] Khoury JE, Kaur H, Gonzalez A (2021). Parental Mental Health and Hostility Are Associated With Longitudinal Increases in Child Internalizing and Externalizing Problems During COVID-19. Front Psychol.

[19] Racine N (2021). Global Prevalence of Depressive and Anxiety Symptoms in Children and Adolescents During COVID-19: A Meta-analysis. JAMA Pediatr.

[20] Bignardi G (2021). Longitudinal increases in childhood depression symptoms during the COVID-19 lockdown. Arch Dis Child.

[21] Lehmann S, Skogen JC, Sandal GM, Haug E, Bjørknes R (2022). Emerging mental health problems during the COVID-19 pandemic among presumably resilient youth -a 9-month follow-up. BMC Psychiatry.

[22] Oliveira TDO et al. Children’s behavioral problems, screen time, and sleep problems’ association with negative and positive parenting strategies during the COVID-19 outbreak in Brazil. Child Abuse Negl 2021;105345. 10.1016/j.chiabu.2021.105345. 10.1016/j.chiabu.2021.105345 PMC9221927 34625278

[23] McArthur BA, Browne D, Racine N, Tough S, Madigan S (2022). Screen Time as a Mechanism Through Which Cumulative Risk is Related to Child Socioemotional and Developmental Outcomes in Early Childhood. Res Child Adolesc Psychopathol.

[24] Tamana SK (2019). Screen-time is associated with inattention problems in preschoolers: Results from the CHILD birth cohort study. PLoS One.

[25] Tandon PS, Zhou C, Johnson AM, Gonzalez ES, Kroshus E (2021). Association of children’s physical activity and screen time with mental health during the COVID-19 pandemic. JAMA Netw Open.

[26] Jericho M, Elliott A (2020). Youth health in a digital world: approaching screen use in clinical practice. Clin Child Psychol Psychiatry.

[27] Paulus MP (2019). Screen media activity and brain structure in youth: Evidence for diverse structural correlation networks from the ABCD study. Neuroimage.

[28] Fors PQ, Barch DM (2019). Differential Relationships of Child Anxiety and Depression to Child Report and Parent Report of Electronic Media Use. Child Psychiatry Hum Dev.

[29] Guerrero MD, Barnes JD, Chaput J-P, Tremblay MS (2019). Screen time and problem behaviors in children: exploring the mediating role of sleep duration. Int J Behav Nutr Phys Act.

[30] Hisler G, Twenge JM, Krizan Z (2020). Associations between screen time and short sleep duration among adolescents varies by media type: evidence from a cohort study. Sleep Med.

[31] Liu M, Wu L, Yao S (2016). Dose–response association of screen time-based sedentary behaviour in children and adolescents and depression: a meta-analysis of observational studies. Br J Sports Med.

[32] Kim S (2020). Differential associations between passive and active forms of screen time and adolescent mood and anxiety disorders. Soc Psychiatry Psychiatr Epidemiol.

[33] Eirich R (2022). Association of Screen Time With Internalizing and Externalizing Behavior Problems in Children 12 Years or Younger: A Systematic Review and Meta-analysis. JAMA Psychiat.

[34] Neville RD, McArthur BA, Eirich R, Lakes KD, Madigan S (2021). Bidirectional associations between screen time and children’s externalizing and internalizing behaviors. J Child Psychol Psychiatr.

[35] Olive LS (2022). Child and Parent Physical Activity, Sleep, and Screen Time During COVID-19 and Associations With Mental Health: Implications for Future Psycho-Cardiological Disease?. Front Psychiatry.

[36] Li X (2021). Screen Use and Mental Health Symptoms in Canadian Children and Youth During the COVID-19 Pandemic. JAMA Netw Open.

[37] Arundell L, Parker K, Timperio A, Salmon J, Veitch J (2020). Home-based screen time behaviors amongst youth and their parents: familial typologies and their modifiable correlates. BMC Public Health.

[38] Barroso NE, Mendez L, Graziano PA, Bagner DM (2018). Parenting Stress through the Lens of Different Clinical Groups: a Systematic Review & Meta-Analysis. J Abnorm Child Psychol.

[39] Kochanova K, Pittman LD, McNeela L (2022). Parenting Stress and Child Externalizing and Internalizing Problems Among Low-Income Families: Exploring Transactional Associations. Child Psychiatry Hum Dev.

[40] Shutzman B, Gershy N (2023). Children’s excessive digital media use, mental health problems and the protective role of parenting during COVID-19. Comput Hum Behav.

[41] Adams E, Smith D, Caccavale LJ, Bean MK. Parents are stressed! Patterns of parent stress across COVID-19. 2020. https://www.researchsquare.com/article/rs-66730/v2 .10.21203/rs.3.rs-66730/v2. 10.3389/fpsyt.2021.626456 PMC8060456 33897489

[42] Hartshorne JK et al. Screen time as an index of family distress. 2020. https://osf.io/zqc4t . 10.31234/osf.io/zqc4t.

[43] Frick PJ (1991). Alabama Parenting Questionnaire.

[44] Shelton KK, Frick PJ, Wootton J (1996). Assessment of parenting practices in families of elementary school-age children. J Clin Child Psychol.

[45] Cohen S, Kamarck T, Mermelstein R (1983). A Global Measure of Perceived Stress. J Health Soc Behav.

[46] Abidin, R. Parenting stress index: professional manual (3rd ed.). Odessa: Psychological Assessment Resources; 1995.

[47] Goodman R (1997). The strengths and difficulties questionnaire: a research note. J Child Psychol Psychiatry.

[48] Davé S, Nazareth I, Senior R, Sherr L (2008). A comparison of father and mother report of child behaviour on the strengths and difficulties questionnaire. Child Psychiatry Hum Dev.

[49] Burkart S (2022). Impact of the COVID-19 pandemic on elementary schoolers’ physical activity, sleep, screen time and diet: a quasi-experimental interrupted time series study. Pediatr Obes.

